# Health service access and utilization among Syrian refugees in Jordan

**DOI:** 10.1186/s12939-016-0399-4

**Published:** 2016-07-14

**Authors:** Shannon Doocy, Emily Lyles, Laila Akhu-Zaheya, Ann Burton, Gilbert Burnham

**Affiliations:** Johns Hopkins Bloomberg School of Public Health, 615 N Wolfe St, Suite E8132, Baltimore, MD 21205 USA; Jordan University of Science and Technology School of Nursing, Irbid, Jordan; United Nations High Commissioner for Refugees, Amman, Jordan

**Keywords:** Syria, Jordan, Refugee, Humanitarian assistance, Health services

## Abstract

**Background:**

The influx of Syrian refugees into Jordan presents an immense burden to the Jordanian health system. Changing lifestyles and aging populations are shifting the global disease burden towards increased non-infectious diseases including chronic conditions, co-morbidities, and injuries which are more complicated and costly to manage. The strain placed on health systems threatens the ability to ensure the health needs of both refugees and host country populations are adequately addressed. In light of the increasing challenges facing host governments and humanitarian actors to meet health needs of Syrian refugees and affected host communities, this study was undertaken to assess utilization of health services among Syrian refugees in non-camp settings.

**Methods:**

A survey of Syrian refugees in Jordan was undertaken in June 2014 to characterize health seeking behaviors and issues related to accessing care. A cluster design with probability proportional to size sampling was used to attain a nationally representative sample of 1550 non-camp Syrian refugee households. Differences in household characteristics by geographic region, facility type, and sector utilized were examined using chi-square and *t*-test methods.

**Results:**

Care-seeking was high with 86.1 % of households reporting an adult sought medical care the last time it was needed. Approximately half (51.5 %) of services were sought from public sector facilities, 38.7 % in private facilities, and 9.8 % in charity/NGO facilities. Among adult care seekers, 87.4 % were prescribed medication during the most recent visit, 89.8 % of which obtained the medication. Overall, 51.8 % of households reported out-of-pocket expenditures for the consultation or medications at the most recent visit (mean US$39.9, median US$4.2).

**Conclusions:**

Despite high levels of care-seeking, cost was an important barrier to health service access for Syrian refugees in Jordan. The cessation of free access to health care since the time of the survey is likely to have worsened health equity for refugees. Dependence of refugees on the public facilities for primary and specialist care has placed a great burden on the Jordanian health system. To improve accessibility and affordability of health services in an equitable manner for both refugees and Jordanian host communities, strategies that should be considered going forward include shifting resources for non-communicable diseases and other traditional hospital services to the primary level and creating strong health promotion programs emphasizing prevention and self-care are strategies.

## Background

Crises in recent decades have seen a shift in displaced populations settling in more non-camp settings in middle-income countries [1]. The protracted extent of many of these situations necessitates health responses focused on greater integration of refugees into host country systems rather than the establishment of parallel systems of refugee assistance [2–4]. The changing nature of displacement also carries implications for equitable prioritization and provision of health care and other services for refugees. Compounded by global demographic and epidemiologic transitions, a changing burden of disease further requires such systematic redesign of assistance modalities in order to ensure adequate accessibility and coverage of health services and, in turn, health outcomes for refugees [5].

While neonatal and infectious diseases remain the leading cause of excess mortality in low-income settings affected by conflict, changing lifestyles and aging populations are shifting the disease burden towards increased non-infectious diseases including preexisting chronic conditions, multiple co-morbidities, and injuries which are more complicated and costly to manage [6]. The strain placed on health systems threatens the ability to ensure equitable distribution of services across different refugee populations and also between refugees and host country nationals. The capacity of health infrastructure in countries hosting large refugee populations must be improved so that the health needs of both refugees and host country populations are adequately addressed. This requires substantial amounts of resources on the part of host governments, support from the international community, and prioritization of health equity for refugee populations.

Since the start of the crisis in 2011, an estimated 7.6 million Syrians have been displaced within Syria and another 4 million have fled the country, largely to Jordan, Lebanon, and Turkey [7, 8]. A majority (>80 %) of the estimated 630,000 registered Syrian refugees in Jordan reside in host communities rather than in camps and access health services through existing public health services [7, 9]. Before late 2014, Syrian refugees registered with The United Nations High Commissioner for Refugees (UNHCR) in Jordan could receive free primary, secondary and some tertiary health care at public facilities. During this time, co-payments were not required for many primary health services and care at government hospitals was accessible to refugees with referral from public health centers. While refugees currently have access to public sector services in Jordan, in an effort to improve relatively equal access to health services between refugee and host communities, refugees are now required to make co-payments equivalent to those required of uninsured Jordanians. While these costs are still highly subsidized, they often prove more than resource-constrained households can meet. With funding support for international assistance programs waning, even nominal costs for refugees can be a barrier to care [10].

Despite increasing research into health care-seeking and care utilization among Syrian refugees in Jordan, there is a dearth of such research that applies similar methodologies to assess health care-seeking and utilization among the Jordanian host community for comparative purposes. One study conducted in 2014 assessed perceptions of access to health care and tensions between Jordanian host communities and Syrian refugees and found that more Syrians (66 %) than Jordanians (57 %) reported adequate access to health care [11]. In particular, overcrowded health facilities were reported as a main challenge and source of tension by more Jordanians (60 %) than Syrians (39 %). Owing to reported perceptions of inequity in access to services, it is essential to contextualize Syrian refugee health care utilization with previously reported data on utilization by Jordanian host communities.

In light of the increasing challenges facing host governments and humanitarian actors to meet health needs of Syrian refugees, we undertook this study to assess access and utilization of health services among Syrian refugees in non-camp settings in Jordan.

## Methods

A cross-sectional survey of Syrian refugees in Jordan was conducted in June 2014, to characterize health seeking behaviors and better understand issues related to accessing health services. A two-stage cluster survey design with probability proportional to size sampling was used to obtain a nationally representative sample of Syrian refugees living outside of camps. Sample size was determined for key study objectives based on the most conservative prevalence rate estimate of 50 %; calculations assumed 80 % power and a design effect of 2.0 to account for the cluster sample design. The planned sample size was increased to account for the possibility of up to a 10 % non-response rate. The planned sample size was increased from the minimum identified size of 900 households to 1500 households to provide increased precision of point estimates and additional power for the detection of statistically significant differences of >10 % for the comparison of key indicators between sub-national regions.

**Fig. 1:**
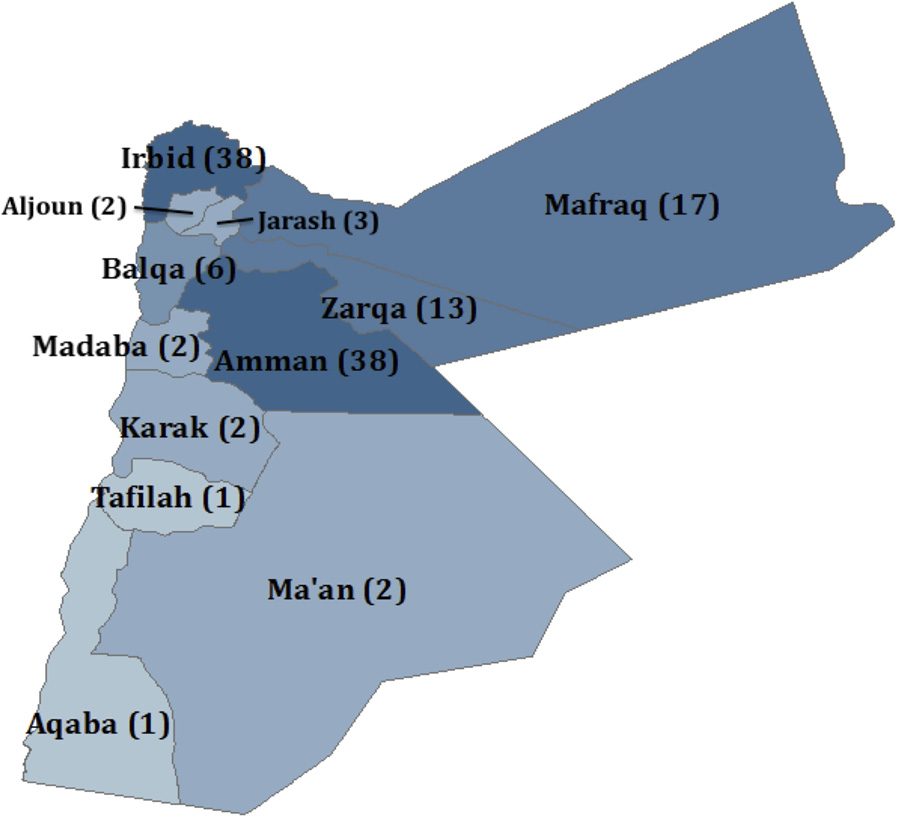
Distribution of study clusters

Given the geographic concentration of Syrian refugees and the low cost of visiting many locations in Jordan due to the country’s small size and good transportation infrastructure, a 125 cluster x 12 household design was chosen. Probability proportional to size sampling was used to assign the number of clusters to sub-districts using UNHCR registration data, assuming that non-registered refugees had similar residence patterns. The final cluster assignment included 38 clusters (30 %) in Amman governorate, 38 clusters (30 %) in Irbid governorate, and 49 clusters (40 %) distributed proportionately in the remaining nine governorates (Fig. 1). In each cluster, UNHCR randomly selected five registered refugee households that were listed as living in that cluster’s assigned sub-district. Households were then telephoned by the study team; the first household that was currently residing in the specified sub-district and agreed to meet with the study team was used as the index household for the cluster. The study team met this household, conducted an abbreviated interview (the results of which were not included in the survey data set to minimize bias towards registered refugees), and enquired about Syrian households living in the vicinity. The household(s) to which the index household referred the interview teams were then interviewed using the complete questionnaire and were included. Household heads and primary caretakers of children were prioritized as respondents and answered questions on behalf of the households and its members. Household members were defined as people who share a dwelling space and share meals, regardless of biological relation; short-term visitors were not counted as household members. At the conclusion of each interview, households were asked for a referral to the nearest Syrian household. This within cluster referral process was used until ten interviews were completed. Only Syrian households arriving in Jordan in 2011 or after were eligible to participate in the survey; however, none of the households approached for interview arrived in Jordan before 2011.

The questionnaire was developed by consensus between partner agencies and focused on health service utilization, access to care, barriers to care seeking, children’s health and vaccination, and chronic medical conditions. The questionnaire was translated to Arabic after which the translation was reviewed and discussed by in-country re-searchers and WHO technical staff, including a Syrian physician, and a consensus translation was agreed upon. Both a pre-pilot test and a formal pilot test were performed. Interviewers underwent two days of classroom training on the questionnaire, e-data collection using tablets, interview techniques, basic principles of human subjects’ protections and sampling methods followed by one day of practical field training. To protect the anonymity of respondents, no information was recorded that could be used to identify the household or individuals and verbal consent was obtained from all respondents. Interviews lasted between 30 and 60 min depending on the household size, number of children and individuals with chronic medical conditions. Data was collected on tablets using the Magpi mobile data platform by DataDyne LLC (Washington, DC). Data was analyzed using Stata 13 (College Station, TX) and Tableau Desktop (Seattle, WA) software packages and employed standard descriptive statistics and methods for comparison of means and proportions. Outliers in hospital cost data were replaced with the highest plausible value in the distribution (i.e., Winsorized). The Stata ‘*svy’* command was used to account for the cluster survey design so that standard errors of the point estimates were adjusted for survey design effects.

The study was approved by ethics committees at the World Health Organization, Jordan University of Science and Technology and Johns Hopkins School of Public Health and was also approved by the Jordanian Ministry of Health.

## Results

A total of 1,634 households were approached to participate in the survey. Of these households, 2.9 % (*n* = 47) were not at home, 0.8 % (*n* = 14) were already interviewed for this survey, and 1.4 % (*n* = 23) declined to be interviewed. The final sample included 1,550 households (with 9,580 household members), which equates to a response rate of 94.7 %.

### Health seeking and service utilization

The primary reasons adult refugees reported needing care included infections or communicable diseases (21.5 %), chronic medical conditions and non-communicable diseases (21.1 %), injuries (9.7 %) and dental care (8.0 %). Approximately half of adults (61.6 %, CI: 58.7–64.5) reported needing medical care within the month preceding the survey. Overall, 86.1 % (CI: 83.6 %–88.2 %) of households reported that medical attention was sought the last time an adult needed medical care. Among the 13.9 % (CI: 11.8–16.4) of households that did not seek care for an adult last time it was needed, the primary reason was cost, where 64.5 % (CI: 56.7–71.6) reported they could not afford to seek medical services. Other reasons included not being sick enough to seek care (6.5 %, CI: 3.6–11.6), not knowing where to go (5.9 %, CI: 3.3–10.3), provider having inadequate medications or equipment (5.3 %, CI: 2.9–9.6 %), still waiting for a scheduled appointment (5.3 %, CI: 2.9–9.6 %) and transportation difficulties (4.1 %, CI: 2.0–8.3). Reasons for needing care, timeframe of most recently needing care, receipt of care and reasons for not seeking care are presented in Table 1. No significant differences in the reason care was needed (*p* = 0.103), the last time care was needed (*p* = 0.192), care seeking rates (*p* = 0.192) or reasons for non-care seeking (*p* = 0.188) were observed by region.

**Table 1:** Health care needs among adult Syrian Refugees in Jordan

	Survey total	
	%	95 CI
Reason for needing health services^b^	*n* = 1212	
Injury	9.7	[8.2,11.5]
Infection/communicable disease^c^	21.5	[19.3,23.9]
Chronic/non-communicable disease	21.1	[18.8,23.6]
Dental care	8.0	[6.5,9.8]
Skin problem	3.9	[2.9,5.3]
Emotional or mental health	1.5	[0.9,2.4]
Gastrointestinal	4.4	[3.3,5.9]
Renal	4.0	[2.9,5.3]
Eye problem	2.3	[1.6,3.2]
Obstetric/Gynecological	6.5	[5.2,8.1]
Joint Pain	5.1	[4.0,6.6]
Other	12.0	[10.2,14.0]
Timeframe when care was last needed^b^		
Less than two weeks ago	37.8	[34.8,40.8]
2 weeks to less than 1 month ago	23.8	[21.4,26.4]
1 month to less than 3 months ago	22.0	[19.7,24.5]
3 months to less than 6 months ago	9.3	[7.8,11.1]
6 months to less than 1 year ago	4.9	[3.8,6.3]
More than 1 year ago	2.1	[1.4,3.2]
Received care last time care was needed^b^		
Yes	86.1	[83.6,88.2]
No	13.9	[11.8,16.4]
Reason for deciding not to seek care^d^	*n* = 169	
Could not afford provider costs	64.5	[56.7,71.6]
No transportation/difficult to access	4.1	[2.0,8.3]
Could not afford transportation costs	1.8	[0.6,5.3]
Equipment or drugs are inadequate	5.3	[2.9,9.6]
Disliked treatment on previous visit(s)	3.0	[1.2,6.9]
Could not take time/other commitments	0.6	[0.1,4.1]
Did not know where to go	5.9	[3.4,10.2]
Not sick enough to seek care	6.5	[3.6,11.6]
Appointment scheduled/still waiting	5.3	[2.8,9.9]
Other	3.0	[1.3,6.8]

^a^No significant differences were observed for any indicators in the table in three way comparisons by region

^b^As percent of cases where it was reported that care was needed in Jordan

^c^Including cough, cold, flu

^d^As percent of cases that did not seek care last time it was needed in Jordan

**Fig. 2:**
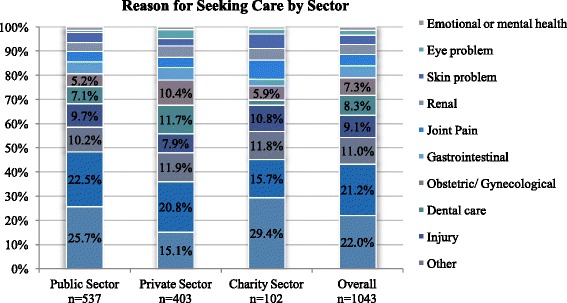
Reason for seeking care by sector

Approximately half (51.5 %, CI: 47.7–55.3) of those in need of attention sought care in public sector facilities including public hospitals (22.9 %), primary health care centers (21.0 %), and comprehensive health centers (7.6 %). Another 38.7 % (CI: 35.3–42.2) sought care in private sector facilities including private hospitals (9.3 %), private clinics (22.0 %), pharmacies (5.4 %), Syrian doctors (1.7 %, CI: 1.1–2.7) and shops or other informal providers (0.3 %). Charity/NGO facilities were used by the remaining 9.8 % (CI: 7.6–12.5) of care seekers, including non-religious charities (7.2 %) and Islamic charities (2.6 %). Differences in adult care seeking location by region were marginally statistically significant (*p* = 0.073); a higher proportion of households in the South (70.7 %) used public sector facilities as compared to in the North (52.9 %) and Central (47.8 %) regions. Differences in the reason for care seeking were marginally statistically significant by region (*p* = 0.077) and statistically significant by sector (*p* < 0.001); differences by sector are summarized in Fig. 2 and presented in detail in Table 2.

**Table 2:** Health service utilization among adult Syrian Refugees in Jordan

	Overall		By sector					
			Public		Private		Charity	
Consultations	%	95 CI	%	95 CI	%	95 CI	%	95 CI
Reason for seeking care^a^	*n* = 1043		*n* = 537		*n* = 403		*n* = 102	
Chronic/non-communicable disease	22	[19.4,24.8]	25.7	[22.1,29.7]	15.1	[11.9,19.1]	29.4	[21.3,39.0]
Infection or communicable disease	21.2	[18.9,23.7]	22.5	[19.1,26.4]	20.8	[17.2,25.1]	15.7	[9.1,25.8]
Other	11.0	[9.2,13.2]	10.2	[7.8,13.4]	11.9	[9.2,15.3]	11.8	[7.0,19.0]
Injury	9.1	[7.4,11.1]	9.7	[7.5,12.4]	7.9	[5.6,11.1]	10.8	[6.2,18.2]
Dental care	8.3	[6.7,10.3]	7.1	[5.1,9.8]	11.7	[8.9,15.1]	2.0	[0.5,7.7]
Obstetric/Gynecological	7.3	[5.8,9.1]	5.2	[3.7,7.4]	10.4	[7.8,13.8]	5.9	[2.7,12.2]
Gastrointestinal	4.9	[3.6,6.6]	5.0	[3.4,7.4]	5.2	[3.3,8.0]	2.9	[1.0,8.7]
Joint Pain	4.6	[3.4,6.2]	4.3	[2.7,6.8]	4.2	[2.7,6.5]	7.8	[4.2,14.2]
Renal	4.2	[3.1,5.8]	3.7	[2.4,5.8]	4.7	[2.9,7.5]	4.9	[2.1,11.0]
Skin problem	3.8	[2.8,5.2]	4.1	[2.7,6.1]	3.0	[1.7,5.3]	5.9	[2.6,12.6]
Eye problem	2.1	[1.4,3.2]	0.9	[0.4,2.2]	3.7	[2.2,6.2]	2.0	[0.5,7.7]
Emotional or mental health	1.4	[0.8,2.5]	1.5	[0.8,2.9]	1.2	[0.5,2.9]	1.0	[0.1,6.5]
Statistical significance			*Three-way regional comparison p < 0.001*					
Location of most recent care in Jordan^a^								
Government primary health care center	21.0	[17.3,25.2]	40.8	[34.8,47.0]		--		--
Government comprehensive center	7.6	[6.0,9.5]	14.7	[11.7,18.4]		--		--
Pharmacy	5.4	[4.0,7.2]		--	13.9	[10.5,18.2]		--
Private Jordanian clinic or doctor	22.0	[19.1,25.1]		--	56.8	[51.3,62.2]		--
Public hospital	22.9	[20.1,26.0]	44.5	[39.0,50.2]		--		--
Private hospital	9.3	[7.5,11.5]		--	24.1	[19.6,29.2]		--
Syrian doctor	1.7	[1.1,2.7]		--	4.5	[2.8,7.1]		--
Islamic charity	2.6	[1.8,3.8]		--		--	26.5	[18.3,36.6]
Non-religious charity	7.2	[5.3,9.6]		--		--	73.5	[63.4,81.7]
Shop or other	0.3	[0.1,0.9]		--	0.7	[0.2,2.3]		--
Statistical significance			*Three-way regional comparison p < 0.001*					
Medications	*n* = 1043		*n* = 537		*n* = 403		*n* = 102	
Prescribed medication during most recent health facility visit^a^	87.4	[85.0,89.5]	85.1	[81.5,88.1]	91.6	[88.5,93.9]	84.3	[75.5,90.4]
Statistical significance			*Three-way regional comparison p < 0.008*					
Able to obtain all medications prescribed during provider visit^b^	*n* = 912		*n* = 457		*n* = 369		*n* = 86	
	89.8	[87.5,91.7]	86.7	[82.4,90.0]	93.8	[90.5,96.0]	89.5	[81.3,94.4]
Statistical Significance			*Three-way regional comparison p < 0.009*					
Reason for not obtaining medication^c^	*n* = 93		*n* = 61		*n* = 23		*n* = 9	
Medication was out of stock (public facility)	51.6	[41.6,61.5]						
Household could not afford the medication	39.8	[30.3,50.1]	Insufficient sample sizes for sector comparison					
Did not know where to get the medication	2.2	[0.5,8.3]						
Others	6.5	[2.1,13.7]						

^a^among cases that received care; ^b^among cases prescribed medication during visit; ^c^among cases not obtaining prescribed medication

### Predictors of care seeking

Results of univariate and multivariate logistic regression analyses for predictors of sector-specific care seeking for adults are presented in Table 3. In the public sector, significant differences in the adjusted odds of care seeking were observed by region and socioeconomic status. Care seekers in the South were 7.80 (CI: 1.83–33.23) times more likely to seek care in the public sector than those in the North. Care seekers in the lowest quartile were most likely to use the public sector and all other quartiles had significantly lower odds of public sector care seeking at the *p* < 0.10 level; at the *p* < 0.05 level of significance, odds of care seeking for the 2nd and top quartiles were 0.35 (CI: 0.16–0.80) and 0.15 (CI: 0.05–0.46), respectively. Both region and socioeconomic status were significant predictors of care seeking in the adjusted model for the private sector, though opposite trends were observed as compared to the public sector. With respect to region, households in Central Jordan had significantly higher odds of care seeking in the private sector as compared to those in the North (2.01, CI: 1.01–3.98). Households in the bottom socioeconomic quartile were least likely to seek care in the private sector; both the 2nd and top quartiles had significantly higher odds of care seeking in the private sector, at 2.68 (CI: 1.20–6.00) and 4.38 (CI: 1.62–11.88), respectively. Finally, in the charity sector, there were no statistically significant differences in adjusted odds of care seeking among the variables assessed, however, it should be noted that the sample size for this model was much smaller which may have contributed to a lack of statistical significance.

**Table 3:** Odds of sector-specific care seeking for adult Syrian Refugees^a^

	Public sector (*n* = 537)						Private sector (*n* = 403)						Charity sector (*n* = 102)					
	Crude OR^*^		*p*-value	Adjusted OR^**^		*p*-value	Crude OR^*^		*p*-value	Adjusted OR^**^		*p*-value	Crude OR^*^		*p*-value	Adjusted OR^**^		*p*-value
Region of residence																		
North	Reference			Reference			Reference			Reference			Reference			Reference		
Central	0.82	(0.60,1.11)	0.203	0.61	(0.29,1.30)	0.200	**1.66**	**(1.24,2.23)**	**0.001**	**2.01**	**(1.01,3.98)**	**0.045**	**0.50**	**(0.28,0.76)**	**0.003**	0.60	(0.24,1.53)	0.283
South	**2.15**	**(1.07,4.35)**	**0.033**	**7.80**	**(1.83,33.23)**	**0.006**	0.76	(0.40,1.43)	0.397	0.27	(0.07,1.05)	0.058	**0.11**	**(0.02,0.78)**	**0.027**	1.00		
Household characteristics																		
Crowding (5+/ sleeping room)	1.13	(0.83,1.54)	0.437	1.87	(0.85,4.14)	0.121	**0.68**	**(0.50,0.92)**	**0.014**	0.63	(0.28,1.41)	0.258	**1.85**	**(1.16,2.96)**	**0.011**	0.60	(0.13,2.74)	0.502
Registered with UNHCR	1.36	(0.91,2.03)	0.130	0.95	(0.34,2.64)	0.916	0.78	(0.54,1.13)	0.188	1.08	(0.38,3.04)	0.881	0.82	(0.43,1.56)	0.549	0.93	(0.18,4.94)	0.934
Household head education (highest level completed)																		
None	Reference			Reference			Reference			Reference			Reference			Reference		
Primary	**0.39**	**(0.16,0.93)**	**0.034**	0.52	(0.18,1.45)	0.208	2.15	(0.78,5.92)	0.136	1.59	(0.51,4.96)	0.423	1.87	(0.42,8.35)	0.410	1.60	(0.37,7.01)	0.525
Preparatory	**0.46**	**(0.19,1.11)**	**0.084**	0.84	(0.27,2.61)	0.760	**2.35**	**(0.90,6.11)**	**0.080**	1.31	(0.42,4.09)	0.633	0.94	(0.18,5.02)	0.939	0.73	(0.14,3.95)	0.716
Secondary or higher	0.81	(0.28,2.29)	0.686	1.78	(0.53,6.01)	0.347	1.01	(0.35,2.92)	0.988	0.50	(0.15,1.67)	0.258	1.71	(0.31,9.38)	0.530	1.14	(0.21,6.11)	0.873
Socioeconomic quartile (by monthly expenditures)																		
Bottom	Reference			Reference			Reference			Reference			Reference			Reference		
2nd	0.73	(0.49,0.19)	0.123	**0.35**	**(0.16,0.80)**	**0.014**	1.21	(0.78,1.88)	0.391	**2.68**	**(1.20,6.00)**	**0.017**	1.41	(0.80,2.49)	0.231	1.51	(0.28,8.12)	0.628
3rd	**0.50**	**(0.35,0.71)**	**<0.001**	0.47	(0.20,1.13)	0.091	**2.03**	**(1.40,2.94)**	**<0.001**	1.89	(0.82,4.38)	0.135	1.08	(0.59,1.97)	0.802	1.62	(0.42,6.18)	0.475
Top	**0.46**	**(0.32,0.67)**	**<0.001**	**0.15**	**(0.05,0.46)**	**0.001**	**2.02**	**(1.34,3.03)**	**0.001**	**4.38**	**(1.62,11.88)**	**0.004**	1.37	(0.74,2.51)	0.311	3.05	(0.50,18.61)	0.224
Year of arrival in Jordan																		
2011–2012	Reference			Reference			Reference			Reference			Reference			Reference		
2013–2014	0.90	(0.71,1.14)	0.387	0.91	(0.41,2.02)	0.818	0.91	(0.70,1.18)	0.482	1.11	(0.53,2.32)	0.785	**1.82**	**(1.19,2.77)**	**0.006**	0.98	(0.33,2.87)	0.965

^a^Care seeking defined as having sought care last time it was needed

^*^ Bold indicates statistically significant (*p* < 0.10) findings

^**^Bold indicates statistically significant (*p* < 0.05) findings

### Access to medicines

Among adult care seekers, 87.4 % (CI: 85.0–89.5) reported being prescribed medication at their most recent visit to a health facility. No significant differences were observed by region (*p* = 0.274) with respect to the proportion of patients receiving a prescription. Significant differences in the proportion of patients receiving a prescription were observed between provider types with the greatest proportion of patients receiving a prescription in private facilities (91.6 %, CI: 88.5–93.9) and the lowest in charity/NGO facilities (84.3 %, CI: 75.5–90.4) (*p* = 0.008). Of those prescribed medication 89.8 % (CI: 87.5–91.7) were able to obtain all of the prescribed medications. Among those that did not access medications, the primary reasons included that the medication was out of stock at the public facility (51.6 %, CI: 41.6–61.5 %) or that the household could not afford the medication (39.8 %, CI: 30.3–50.1); reasons for not obtaining medications were similar across regions (*p* = 0.875).

### Spending on health

Of the 1212 families with adults needing care identified in the survey, 1043 (86 %) sought care or treatment for the adult (Table 4). Among the 1043 families that sought care, half (51.8 %, CI: 47.9–55.6) reported an out-of-pocket payment. The average total out-of-pocket cost per visit among all seeking care was US$39.9 (US$25.6 for consultations, US$14.2 for medications); however, the median values were US$4.2 for total costs and US$0 for consultations and medication. Mean costs account for approximately 6 % (total costs), 3.8 % (consultation costs), and 2.1 % (medication costs) of reported monthly household expenditures and 12.4 % (total costs), 7.9 % (consultation costs), and 4.4 % (medication costs) of reported monthly household income, relatively high proportions for one care visit, particularly for conditions requiring continuous care. There was a statistically significant difference in total out-of-pocket cost per visit by sector as follows: private facilities, US$75.6 (US$50.5 for consultations, US$25.1 for medications); public facilities, US$18.0 (US$11.0 for consultations, US$7.0 for medications); and NGO/Charity facilities, US$14.0 (US$4.8 for consultations, US$9.2 for medications) (*p* < 0.001). Out-of-pocket payment amounts did not vary significantly by type of facility where care was sought (*p* = 0.395) or by geographic region (*p* = 0.215).

**Table 4:** Out-of-pocket payments for consultation fees, medications, and healthcare visit^a^

		Survey total		By facility type^b^						By sector					
				Primary/secondary		Hospital		Pharmacy		Public		Private		NGO/charity	
		Point	95 CI	Point	95 CI	Point	95 CI	Point	95 CI	Point	95 CI	Point	95 CI	Point	95 CI
Among all care seekers		*n* = **1043**		*n* = **573**		*n* = **336**		*n* = **131**		*n* = **537**		*n* = **403**		*n* = **102**	
Total costs	Median	4.2		7.1		0		4.2		0		29.6		0	
	Mean	39.9	[30.3,49.4]	30.4	[22.8,37.9]	67.3	[43.6,91.1]	11.4	[7.7,15.1]	***18.0***	***[9.5,26.5]***	***75.6***	***[53.7,97.5]***	***14.0***	***[4.8,23.1]***
Consultation costs	Median	0		0		0		0		0		14.1		0	
	Mean	25.6	[17.2,34.1]	16.9	[10.8,23.0]	50.2	[28.7,71.7]	1.5	[0.6,2.3]	***11.0***	***[3.7,18.2]***	***50.5***	***[30.7,70.4]***	***4.8***	***[0,11.7]***
Medication costs	Median	0		0		0		4.2		0		14.1		0	
	Mean	14.2	[12.3,16.2]	13.5	[11.2,15.7]	17.2	[13.0,21.3]	9.9	[6.5,13.3]	***7.0***	***[5.1,9.0]***	***25.1***	***[21.4,28.8]***	***9.2***	***[4.3,14.1]***
Among care seekers with any payment		*n* = **1039**		*n* = **314**		*n* = **151**		*n* = **74**		*n* = **145**		*n* = **360**		*n* = **35**	
Total costs	Median	31		35.2		49.3		14.1		28.2		35.2		22.6	
	Mean	77.0	[59.3,94.7]	55.4	[43.0,67.8]	149.8	[101.0,198.6]	20.1	[14.9,25.4]	66.7	[39.1,94.3]	84.7	[60.2,109.1]	40.7	[17.4,63.9]
Consultation costs	Median	9.9		14.1		14.1		0		0		14.1		0	
	Mean	49.5	[33.5,65.6]	30.8	[20.2,41.5]	111.6	[66.4,156.8]	2.6	[1.1,4.0]	40.6	[15.8,65.5]	56.6	[34.3,78.9]	13.9	[0,33.7]
Medication costs	Median	16.9		14.1		28.2		14.1		14.1		16.9		21.1	
	Mean	27.5	[24.3,30.6]	24.6	[21.4,27.7]	38.2	[30.2,46.2]	17.5	[12.7,22.4]	26.1	[20.7,31.4]	28.1	[24.2,32.0]	26.8	[16.1,37.6]
Among households paying for consultation		*n* = **368**		*n* = **257**		*n* = **93**		*n* = **17**		*n* = **60**		*n* = **291**		*n* = **17**	
Consultation costs	Median	21.1		14.1		42.3		7.1		42.3		21.1		5.6	
	Mean	72.7	[49.6,95.8]	***37.7***	***[24.9,50.4]***	***181.2***	***[111.4,251.1]***	***11.2***	***[6.9,15.5]***	98.2	[44.7,151.7]	70.0	[43.1,97.0]	28.5	[0,68.4]
Among households paying for medications		*n* = **473**		*n* = **272**		*n* = **130**		*n* = *n* = **70**		*n* = **125**		*n* = **316**		*n* = **32**	
Medication costs	Median	21.1		21.1		28.2		14.1		21.1		21.1		21.1	
	Mean	31.3	[28.0,34.7]	28.4	[24.9,31.8]	44.4	[36.0,52.7]	18.6	[13.6,23.6]	30.2	[24.3,36.2]	32.0	[27.9,36.1]	29.3	[18.2,40.5]

^**a**^All costs presented in USD; bold italic indicates statistically significant (*p* < 0.05) findings

^b^Private providers are included under primary/secondary and shops reported with pharmacies

**Fig. 3:**
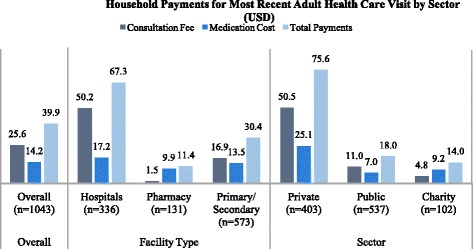
Household payments for most recent adult health care visit by sector (USD)

Details about the total and component costs of treatment (consultations and medications) are provided in Table 4 and Fig. 3. Among the 1043 families who sought care for a sick adult member, 540 reported out-of-pocket payment for consultation, medication, or both (Table 4). Among these families who paid for consultations and/or medications, the average total payment was US$77 (US$49.5 for consultations, US$27.5 for medications). The median values of these payments were as follows: total cost, US$31; consultation cost, US$9.9; and medication cost, US$16.9. There were no statistically significant differences in costs across the three regions, type of facility, or sector among paying families, but generally out-of-pocket payments were lowest in the North, in pharmacies, and in NGO/Charity facilities.

Among the 1043 families who sought care or treatment for a sick adult household member, 368 reported paying for consultation, regardless of whether or not they paid for medications (Table 4). Among these 368 families who paid for consultations, the average and median cost per consultation was US$72.7 and US$21.1, respectively. While there were no statistically significant differences in average consultation cost across regions (*p* = 0.714), differences were statistically significant across types of facilities (*p* = 0.002). The average consultation cost was US$181.2 at hospitals (median = US$42.3), US$37.7 at primary/secondary level facilities (median = $14.1), and US$11.2 at pharmacies (median = US$7.1). Although the differences in average consultation costs among these 296 families was not statistically significant by sector (*p* = 0.151), there were large differences between the average consultation costs at public facilities (US$96.2) and private facilities (US$70.0), compared with NGO/Charity facilities (US$28.5), likely due to the use of public sector hospitals for more serious conditions.

Among the 1043 families who sought care or treatment for a sick adult member, 819 reported receiving medications, of which 473 reported paying for the medications, regardless of whether or not they paid for a consultation (Table 4). Of all refugees receiving medications, 57.8 % (CI: 53.4–62.0) reported paying for the medication. The proportion of refugees incurring any out-of-pocket payment for medication differed significantly across the three sectors as follows: private, 91.3 % (CI: 87.8–93.9); NGO/charity, 41.6 % (CI: 28.5–56.0); and public, 31.6 % (CI: 26.2–37.4) (*p* < 0.001). Among these 473 families who paid for medications, the average cost per medication was US$31.3 (median = US$21.1). The differences in the average cost per medication (among paying families) were not statistically significant across the three regions (*p* = 0.274), the three types of health facilities (*p* = 0.847), or across the three sectors (*p* = 0.829).

### Hospitalizations

In the year preceding the survey, 21.2 % (CI: 18.9–23.6) of households reported one or more hospitalizations of a household member in Jordan for reasons other than childbirth. Households reported an average of 2.0 (CI: 1.7–2.3, median = 1, range = 1–20) hospitalizations in the six months preceding the survey. No significant regional differences were observed in the proportion of households with a hospitalization (*p* = 0.841) or the average number of hospitalizations by region (0.433). The primary reasons for hospitalization included injury (20.7 %, CI: 16.7–25.5), cardiovascular conditions (13.7 %, CI: 10.4–17.8), and respiratory conditions (12.2 %, CI: 8.9–16.6) and digestive conditions (7.0 %, CI: 4.6–10.6); no significant differences were observed by region (*p* = 0.333) or facility type (*p* = 0.438).

Most households reported the most recent hospitalization was in a public sector facility (68.0 %, CI: 61.7–73.7) with the minority using private hospitals (24.7 %, CI: 19.7–30.4) and charity/NGO hospitals (7.3 %, CI: 4.9–10.9). Significant differences in hospital utilization were observed by region, where households in the South were most likely to use public sector facilities (89.5 %) whereas those in Central Jordan more often sought care at private hospitals (31.7 %) and charity/NGO hospitals (11.8 %) (*p* = 0.006) (Table 5). This is likely due to an over-supply of private hospital beds in Amman and the overall disproportionate distribution of private hospitals throughout the country with 77 % of all private hospitals located in the Central region and 64 % in Amman alone [12, 13]. The average length of hospitalization was 5.9 days (CI: 4.5–7.2, median = 2, range 1–120) and there were no significant differences in length of stay by sector (*p* = 0.339). Significant differences were observed in length of stay by region. Average hospital stay was longest in the North at 6.6 days (CI: 4.2–9.1, median = 3, range 1–120) as compared to 5.4 days (CI: 4.0–6.9, median = 2, range 1–80) in Central Jordan and 3.2 days in the South (CI: 1.5–4.8, median = 2, range 1–16) (*p* = 0.04).

**Table 5:** Hospitalizations in Jordan among Syrian Refugees

		Survey total (*n* = 1550)		By region						By sector					
				North (*n* = 728)		Central (*n* = 745)		South (*n* = 77)		Public (*n* = 223)		Private (*n* = 81)		Charity (*n* = 24)	
		Point	[95 CI]	Point	[95 CI]	Point	[95 CI]	Point	[95 CI]	Point	[95 CI]	Point	[95 CI]	Point	[95 CI]
Households with any hospitalizations in the past year (%)		21.2	[18.9,23.6]	20.3	[17.0,24.1]	21.6	[18.7,24.9]	24.7	[14.7,38.4]		----		----		----
Statistical significance				*Three-way regional comparison p = 0.841*											
Number of hospitalizations in Jordan in the past six months	Median	1		1		1		1			----		----		----
	Mean	2.0	[1.7,2.3]	1.9	[1.5,2.2]	2.1	[1.7,2.5]	2.1	[0.8,3.4]		----		----		----
Statistical significance				*Three-way regional comparison p = 0.433*											
Reason for most recent hospitalization^a^		*n* = 328		*n* = 148		*n* = 161		*n* = 19		*n* = 223		*n* = 81		*n* = 24	
Injury		20.7	[16.7,25.5]	22.3	[16.2,29.9]	19.3	[14.0,25.9]	21.1	[7.9,45.4]	20.2	[15.4,26.0]	19.8	[12.0,30.8]	29.2	[15.2,48.6]
Cardiovascular		13.7	[10.4,17.8]	13.5	[8.7,20.5]	14.3	[9.8,20.3]	10.5	[4.8,21.5]	14.8	[10.7,20.2]	12.3	[7.1,20.7]	8.3	[2.2,27.3]
Respiratory		12.2	[8.9,16.6]	16.2	[10.5,24.2]	7.5	[4.4,12.3]	21.1	[11.9,34.5]	14.8	[10.3,20.8]	4.9	[1.8,12.6]	12.5	[3.9,33.2]
Digestive		7.0	[4.6,10.6]	8.1	[4.5,14.2]	6.8	[3.7,12.2]	0		5.8	[3.3,10.2]	8.6	[4.3,16.8]	12.5	[3.9,33.2]
Genitourinary		7.0	[4.7,10.3]	8.1	[4.6,13.8]	6.2	[3.4,11.0]	5.3	[1.0,24.3]	7.2	[4.4,11.5]	7.4	[3.2,16.3]	4.2	[0.6,23.4]
Infection or other acute illness		7.0	[4.6,10.5]	5.4	[2.3,11.9]	8.7	[5.4,13.7]	5.3	[1.1,21.8]	7.6	[4.6,12.4]	7.4	[3.5,15.2]	0	
Cancer/neoplasm		3.4	[1.9,6.0]	2.7	[1.0,7.0]	3.1	[1.3,7.3]	10.5	[2.8,32.7]	2.7	[1.2,5.9]	6.2	[2.5,14.5]	0	
Other		29.0	[23.9,34.6]	23.6	[17.5,31.1]	34.2	[26.5,42.8]	26.3	[12.1,48.1]	26.9	[21.4,33.2]	33.3	[23.0,45.5]	33.3	[18.3,52.8]
Statistical significance				*Three-way regional comparison p = 0.334*											
Location of most recent hospitalization^a^															
Public hospital		68.0	[61.7,73.7]	77.7	[70.6,83.5]	56.5	[47.5,65.1]	89.5	[71.4,96.7]		----		----		----
Private hospital		24.7	[19.7,30.4]	18.9	[13.6,25.7]	31.7	[23.9,40.7]	10.5	[3.3,28.6]		----		----		----
Charity/NGO hospital		7.3	[4.9,10.9]	3.4	[1.5,7.5]	11.8	[7.5,18.0]	0			----		----		----
Statistical significance				*Three-way regional comparison p = 0.006*											
Reason for selecting hospital^a^															
Affordable cost		41.8	[36.2,47.5]	37.2	[28.8,46.4]	45.3	[37.6,53.3]	47.4	[33.2,62.0]	42.2	[35.7,48.9]	32.1	[22.5,43.4]	70.8	[48.8,86.1]
Emergency		25.6	[21.1,30.7]	25.0	[18.3,33.2]	24.8	[19.1,31.7]	36.8	[21.2,55.8]	27.4	[21.8,33.8]	23.5	[14.7,35.3]	16.7	[6.2,37.9]
Referred by doctor		14.0	[10.3,18.8]	21.6	[15.3,29.6]	8.1	[4.4,14.5]	5.3	[0.7,29.1]	16.1	[11.4,22.4]	11.1	[5.5,21.1]	4.2	[0.6,25.1]
Close to place of residence		9.5	[6.6,13.3]	8.8	[5.1,14.7]	10.6	[6.4,16.9]	5.3	[1.1,21.8]	10.8	[7.2,15.8]	7.4	[3.1,16.9]	4.2	[0.6,25.1]
Like staff/treatment quality		4.6	[2.7,7.6]	4.1	[1.6,9.9]	5.0	[2.6,9.2]	5.3	[0.7,29.1]	1.3	[0.4,4.1]	13.6	[7.8,22.5]	4.2	[0.6,25.1]
Other		4.6	[2.7,7.8]	3.4	[1.5,7.5]	6.2	[3.1,12.1]	0		2.2	[0.9,5.3]	12.3	[6.8,21.4]	0	
Statistical significance				*Three-way regional comparison p = 0.243*						Three-way sectoral comparison *p* < 0.001					
Hospitalization length (days)	Median	2		3		2		2		2		2		3	
	Mean	5.9	[4.5,7.2]	6.6	[4.2,9.1]	5.4	[4.0,6.9]	3.2	[1.5,4.8]	5.1	[3.7,6.5]	7.4	[3.7,11.1]	7.5	[3.4,11.5]
Statistical significance				*Three-way regional comparison p = 0.179*						*Three-way sectoral comparison p = 0.140*					
Paid for hospitalization^a^		22.3	[17.6,27.7]	14.9	[9.8,21.9]	31.1	[24.2,38.9]	5.3	[0.7,29.1]	15.7	[11.1,21.8]	40.7	[30.1,52.3]	20.8	[9.0,41.2]
Statistical significance				*Three-way regional comparison p = 0.007*						*Three-way sectoral comparison p < 0.001*					
Cost to household for visit (US Dollars)	Median	0		0		0		0		0		0		0	
	Mean	82.0	[53.9,110.2]	59.6	[21.0,98.2]	110.9	[68.3,153.5]	12.0	[−10.7,34.7]	37.5	[17.7,57.3]	207.0	[118.3,295.6]	74.2	[0,193.1]
Statistical significance				*Three-way regional comparison p = 0.440*						*Three-way sectoral comparison p < 0.006*					
Cost to household for visit (US Dollars)^b^	Median	171		181		171		228		114		285		57	
	Mean	368.6	[276.7,460.4]	400.9	[210.7,591.1]	357.2	[253.3,461.0]	228.0	[228.0,228.0]	238.8	[148.3,329.4]	508.0	[346.6,669.4]	356.4	[0,848.7]
Statistical significance				*Three-way regional comparison p = 0.248*						*Three-way sectoral comparison p < 0.001*					

^a^As percent of households with a hospitalization in Jordan in the past six months

^b^Among households that paid for hospitalization

Out-of-pocket payments for the most recent hospitalization are presented in Table 5. In total, 22.3 % (CI: 17.6–27.7) of households reported on an out-of-pocket payment for the most recent hospitalization. Significant differences in the proportion of hospitalizations with out-of-pocket payments were observed by region (*p* = 0.007) and by hospital sector (*p* < 0.001). Out-of pocket-payments for hospitalizations were reported by 31.1 % (CI: 24.2–38.9) of households in Central Jordan as compared to 14.9 % (CI: 9.8–21.9) in the North and 5.3 % (CI: 0.7–29.1) in the South; this is likely related to the different car e seeking patterns where households in Central Jordan were least likely to seek care at a public hospital. Differences in the proportion of hospitalizations with out-of-pocket payments by hospital sector were statistically significant and as follows: private sector, 40.7 % (CI: 30.1–52.3); charity/NGO sector, 20.8 % (CI: 9.0–41.2); and public sector, 15.7 % (CI: 11.1–21.8) (*p* < 0.001). The average out-of-pocket cost to the household for the most recent hospitalization was US$82.0 (CI: 53.9–110.2, median = 0, range 0–1,454) or 12.3 % of reported monthly household expenditures and 25.5 % of reported monthly household income. Mean out-out-of-pocket payments for the most recent hospitalization by provider sector for all households were statistically significant and as follows: private sector, US$207.0 (CI: 118.3–295.6, median = 0); charity/NGO, US$74.2 (CI: 0–193.1, median = 0); and public sector, US$37.5 (CI: 17.7–57.3, median = 0) (*p* = 0.006). Among households with out-of-pocket payments only, mean payment amounts were US$508.0 (CI: 346.6–669.4, median = 285) in private hospitals, US$356.4 (CI: 0–848.7, median = 57) at charity/NGO hospitals, and US$238.8 (CI: 148.3–329.4, median = 114) at public hospitals (*p* = 0.068).

## Discussion

The high care utilization amongst adult Syrian refugees in Jordan reflects a mixed picture of communicable and infectious disease as well as injuries. While this population makes half of their clinic visits for infectious or communicable diseases, non-communicable diseases are an equally common reason to seek medical care. This survey found that 43.4 % of Syrian refugee households reported one or more household members were previously diagnosed with chronic health condition and a similar survey by UNHCR reported that 39.8 % of Syrian refugee households reported a member with a chronic health condition [14, 15].

The health patterns observed among refugees in Jordan reflect earlier observations of the health risks and health status of Syrians. The consequences of the demographic transition with increasing non-communicable disease burden associated with longer life expectancy and changes from traditional life styles are well documented in previous studies of Iraqi refugees in Jordan and Syria, and is consistent with regional patterns [14, 16]. One study among adults in Aleppo found a 56.9 % prevalence of cigarette smoking among men and 17.0 % among women [17]. A self-rated health status survey in Aleppo in 2004 found 9.1 % of Syrians rated their health as poor, and these were predominantly women [18]. Low levels of physical activity were common in all age groups, but especially among women. The 2004 Aleppo Household Survey found body mass index to steadily increase with age. Obesity was present in 82 % of women over age 45, and obesity tended to be more common in Aleppo than among many of the neighboring countries [19]. While there has been much concern about the demands of non-communicable diseases on the health system, communicable diseases requiring prolonged treatment such as tuberculosis and cutaneous leishmaniosis have dramatically higher rates among refugees than among Jordanians [20].

Injury was the most common cause of hospitalization and reflects the high burden of conflict related injuries. More information about the nature of adult injuries among refugees could guide development of an injury prevention program. Although regulations affecting employment of refugees in Jordan are not well defined, many refugees work informally in low wage labor positions which is inherently more dangerous than the formal employment sector [21]. There are risks of physical dangers, and in some sectors, additional risks such as increased chemical exposure in the agricultural sector. Recent estimates suggest that approximately 10 % of refugee teenage boys were in school and that many are taking part in the informal labor force, puts child refugees at considerable risk [16]. A 2014 study of injuries in Baghdad found that falls were a common source of injuries, most occurring at home [22]. It is likely that the pattern in Jordan is similar as refugees tend to live in crowded, poorly maintained accommodations on the edges of the urban areas [23]. Burns have also been identified as a common injury of concern among Syrians [24].

The frequent visits made by refugees to health facilities suggest access to care at the time of the survey was relatively good. Costs rather than distances were reported as the major barriers. The cost barrier was reflected in the 2014 CARE International study of urban households that found 14 % of households had an unmet health need at the time of the interview [14]. With the November 2014 withdrawal of free access to health services to refugees in Jordan this will become even more of a barrier. In the 2015 UNHCR Health Access Survey found that 86.6 % of households that needed care within the month preceding the survey sought care and that despite subsidies, cost was the primary barrier to receiving needed services which was reported by 36 % of non-careseekers [15]. Some health agencies are piloting cash assistance to facilitate access to health services. This will be used initially for more predictable costs such as antenatal care, delivery, and postnatal care but could extend to aspects of care for key non-communicable diseases if the pilot is successful. Differing characteristics and vulnerabilities among refugee sub-populations and between refugee and host-country populations present both an opportunity and challenge to develop and implement policies and programs that benefit all groups in an equitable manner. Special concern needs to be paid to those living in informal shelters who already have difficulty accessing public services and are more reliant on humanitarian assistance [21]. Moreover, changes in assistance to refugees must also consider the implications for host communities and their perceptions of fairness and equity. Vulnerable host community populations must not be forgotten in the face of growing system-wide demands from an increasing refugee population and frequent tensions between the refugees and Jordanian nationals. A 2014 study of health care and tensions between refugees and host communities in Jordan found that 26 % of Jordanian and 21 % of Syrian refugee respondents reported that perceived unequal access to health services was a main source of tension between the two communities [11]. Unlike refugees, Jordanians not seeking care in previous studies most often cited that the reason for this was home medication treatment of their illness (55 % of non-careseekers) with only 11 % citing cost as the primary barrier to care [25].

Health care utilization patterns among Jordanians in previous studies display differences in care-seeking between refugees and host Jordanians. The 2000 Jordan Healthcare Utilization and Expenditure Survey reported that 63 % of Jordanians reporting an illness in the 14 days preceding the survey, substantially lower than 86.1 % of refugees in our survey that reported receiving needed care though it is worth noting that the reference period in our survey (one year) was much longer than that of the 2000 survey of Jordanians (14 days) and may reduce the comparability of these figures [25]. While differences in care-seeking were observed, sector of care receipt was similar between refugees and Jordanians. A 2009 analysis of the 2000 Jordan Healthcare Utilization and Expenditure Survey reported that overall, Jordanian careseekers received care most often in the Ministry of Health’s public sector (51.4 % as compared to 51.5 % of refugees) with a lower proportion of Jordanians utilizing the private sector (42.1 % of Jordanian careseekers as compared to 38.7 % of refugee households included in our survey) [26]. According to the 2000 survey report, Jordanian care-seekers most frequently reported seeking care at government primary health care centers (33.8 %), markedly higher than the 21.0 % of refugees observed in our survey; however, this gap in public sector care utilization is balanced with 9.7 % of Jordanians seeking care in public hospitals as compared to 22.9 % of Syrian refugees [25]. Private clinics were utilized by 29 % of Jordanians and 22.0 % of Syrian refugees. Not surprisingly, charity/NGO facilities were more commonly reported by Syrian refugees (9.8 %) as compared to Jordanians (3.5 %).

While recent comprehensive data on out-of-pocket health care costs for Jordanians that is comparable to our survey results is not available, cost differences for insured Jordanians may explain the increased use of private facilities as compared to Syrian refugees. Results from previous analyses of the influence of insurance coverage on utilization of health services among Jordanians are mixed. Overall, health insurance coverage did not appear to increase health care utilization; however, in Jordan’s heterogeneous health financing context, specific insurance programs were found to increase the probability of Jordanians seeking care when ill [27]. While changes to refugee health care subsidization begin to bridge the care-seeking gap between refugees and Jordanians, differences observed in health care utilization for Jordanians based on insurance coverage under various programs indicate that more uniform health financing of health care for all Jordanians may more uniformly explain Jordanian care-seeking integration of Syrian refugee utilization.

Providing health care for refugees is a large burden on Jordan’s Ministry of Health. High rates of care seeking at hospitals and hospitalizations could reflect the seriousness of the condition and the high burden of non-communicable diseases which are more complex and often require specialist care. However, efforts should be made to encourage refugees to first utilize primary care facilities, and provide referral services for more complex conditions to hospitals when necessary. Though potentially difficult to implement, this will reduce the burden on already overstretched secondary level facilities and the costs of refugee health care. Such a priority shift may also have implications on equitable utilization of health services in populations settled in more remote areas that are unable to access expensive secondary or tertiary facilities. Additionally, relieving some of the burden on the Jordanian health system will likely ameliorate some of the barriers that have increasingly pushed host communities away from necessary health care seeking.

The reasons for the frequent use of private facilities requires further enquiry. While this may be perceived as an inefficient use of refugee resources when care of an equal quality is available in the NGO and public sector at little cost, it does reduce the burden on Ministry of health facilities. Private facilities often provide a flexibility that the public sector does not have, however, with much higher out-of-pocket costs to refugee careseekers. A general concern is that a good quality care is received, whatever the source chosen. Encouraging facilities to use standard protocols for treatment of NCDs, either those from the Jordan Ministry of Health or the World Health Organization is an important step. Health promotion programs can help improve health seeking behavior as well as build knowledge of home treatment for simple conditions such as upper respiratory infections, leading to even small improvements in refugee health status and reductions in the burden currently faced by the Jordanian health system.

The costs for clinical services for refugees borne by the Ministry of Health, UNHCR, and NGOs is greater than in other crises where non-communicable diseases account for a smaller portion of the burden of disease among refugees. Important in this crisis is the development of health promotional programs specifically directed at refugees for risk recognition and control measures for refugees with non-communicable diseases. In other situations, creating targeted clinical and diagnostics services for patients with hypertension, diabetes, and cardiovascular disease has helped improve the disease control [28]. Establishing these specifically for refugees and providing support and health education services may help bring long term costs down while improving the quality of care for persons with these conditions.

### Study limitations

While every attempt was made to create a robust study design and implement it with care, assessments have limitations. Our reliance on UNHCR registration data may have resulted in sampling bias if the geographic distribution of registered and unregistered households differed. The within clusters, if refugee households referred interviewers to acquaintances rather than the nearest household, as requested, bias could be introduced. The use of small clusters size may have reduced within-cluster similarities and the associated design effect. Replacement sampling, which was done for logistical purposes, also could contribute to bias if there are systematic differences between households where no one was at home compared with those interviewed. Finally, interviews were conducted by Jordanians which could have resulted in a higher refusal rate, hesitance or influence on the part of Syrian refugees in responding to certain questions than if interviews had been conducted by Syrians.

## Conclusions

Syrian refugees in non-camp settings in Jordan have difficulties accessing health services principally because of costs. This barrier is likely to worsen following the 2014 transition from free to subsidized health services and the gradual deterioration of economic status that occurs in many refugee households as a result of prolonged displacement. The dependence of refugees predominantly on the public sector for primary and specialist care has placed a great burden on the Jordanian health sector. Increasing co-pay for the public services and a shift toward utilization of the private sector services will likely decrease refugee access to services. Alternative strategies can focus on moving more resources for non-communicable diseases and other traditional hospital services to the primary care level, creating refugee-focused services and a strong health promotion program emphasizing prevention and more self-care and home management of illness. These efforts will not only benefit refugees but also reduce strain and financial burden on the health system, freeing resources to commit to prioritizing equitable provision of care between and within both refugee and host country national populations. Utilizing more allied health professionals and auxiliary staff at the primary care and community level may also decrease heath costs.

## Abbreviations

NGO, non-governmental organization; UNHCR, United Nations High Commissioner for Refugees
